# Effect of pneumococcal conjugate vaccine introduction on childhood pneumonia mortality in Brazil: a retrospective observational study

**DOI:** 10.1016/S2214-109X(18)30455-8

**Published:** 2019-01-22

**Authors:** Cynthia Schuck-Paim, Robert J Taylor, Wladimir J Alonso, Daniel M Weinberger, Lone Simonsen

**Affiliations:** a Sage Analytica, Portland, ME, USA; b Department of Genetics and Evolutionary Biology, University of São Paulo, São Paulo, Brazil; c Department of Epidemiology of Microbial Diseases, Yale University School of Public Health, New Haven, CT, USA; d Department of Global Health, George Washington University, Washington, DC, USA; e Department of Science and Environment, Roskilde University, Roskilde, Denmark

## Abstract

**Background:**

Understanding the real-world effect of pneumococcal conjugate vaccines (PCVs) on pneumonia mortality is crucial because of the expectation that increased PCV use will substantially reduce the burden of pneumonia deaths in children younger than 5 years. However, few post-vaccine introduction studies have estimated the benefits of PCV use on childhood mortality and results have been inconsistent. Therefore, we set out to assess the effect of introduction of ten-valent pneumococcal conjugate vaccine (PCV10) on pneumonia mortality in children in Brazil.

**Methods:**

In this retrospective observational study, we used publicly available mortality data of children aged 3–59 months in Brazil. We separated data by age group (3–11 months, 3–23 months, and 3–59 months) and stratified data by three different socioeconomic factors of Brazilian municipalities (in 2010): Human Development Index, proportion of children living in extreme poverty, and proportion of mothers with no primary education. We first examined long-term trends in childhood pneumonia mortality in Brazil (from 1980 to 2014). We then assessed the effect of PCV10—introduced in Brazil in 2010—both nationally and in municipalities stratified by socioeconomic status, with a synthetic control approach as our primary analytical method.

**Findings:**

Between 1980 and 2010, a period during which Brazil's Human Development Index rose substantially, national pneumonia mortality in children younger than 5 years decreased from about 150 to 15 deaths per 100 000 children younger than 5 years. Despite rapid uptake of PCV10 after its introduction in 2010, we observed a further vaccine-associated decline of about 10% in national childhood pneumonia mortality with our primary analytical method, with a high degree of uncertainty in the estimates. We observed larger reductions in municipal childhood pneumonia mortality in all three age groups (3–11 months, 3–23 months, and 3–59 months) in municipalities with a high percentage of extreme childhood poverty and mothers with no primary education, with the largest decrease observed in children aged 3–23 months in municipalities with low maternal education (24%, 95% credible interval 7–35).

**Interpretation:**

The large reduction observed from 1980 to 2010 in national pneumonia mortality in children younger than 5 years underscores that improvements in nutrition, hygiene, education, and health care have an important role in reducing pneumonia mortality. Although the PCV-associated reduction in childhood pneumonia mortality at the national level was modest, we found that PCV led to larger reductions in low-income municipalities. Similarly, large benefits might occur when PCVs are introduced in other low-income settings.

**Funding:**

Bill & Melinda Gates Foundation and National Institute of Allergy and Infectious Diseases.

## Introduction

Pneumonia, despite substantial progress in prevention and treatment, is still the leading cause of death due to infectious diseases in children younger than 5 years, causing between 812 000 and 1·1 million deaths in children annually.^1^ More than half of all global pneumonia deaths in children are thought to be caused by *Streptococcus pneumoniae* (the pneumococcus),^2^ a bacterial pathogen that also causes invasive pneumococcal disease.

Pneumococcal conjugate vaccines (PCVs) have been introduced globally to reduce the burden of morbidity and mortality due to pneumococcus. The first formulation, a seven-valent PCV (PCV7), was introduced in 2000. Higher valency versions are now in use, covering ten (PCV10) and 13 (PCV13) serotypes. Administered to infants in a multidose schedule, PCVs are included in the national immunisation programmes of 135 countries.^3^ PCVs are expensive and could increase total costs of the WHO Expanded Programme of Immunisation by 60–80%.^4^ Thoroughly understanding the real-world benefits of PCVs is, therefore, doubly important.

Post-introduction studies of effectiveness have shown that PCVs reduce hospitalisations for invasive pneumococcal disease and pneumonia and provide indirect (herd) protection to unvaccinated older children and adults in highly vaccinated populations.^5^, ^6^ The vaccine reduces carriage of covered serotypes throughout the population and, in some countries, vaccine serotypes have been almost eliminated.^7^ Invasive pneumococcal disease caused by pneumococcal serotypes not covered by the vaccine generally increases after vaccine introduction. Despite this replacement, a literature review^7^ found a net benefit against invasive pneumococcal disease in children, whereas, in some adult age categories, increases in non-vaccine serotypes completely offset invasive pneumococcal disease declines in vaccine-targeted serotypes.

**Research in context**

**Evidence before this study**

Since 2000, pneumococcal conjugate vaccines (PCVs) have been introduced in more than 100 countries to reduce the burden of pneumococcal disease. Post-marketing effectiveness studies have shown that PCVs reduce hospitalisations for invasive pneumococcal disease and pneumonia. However, organisations charged with implementing PCV programmes expect most to achieve a reduction in childhood pneumonia mortality. For example, WHO has stated that PCVs could reduce global pneumonia mortality in children by up to 30% and thus, should be a priority in all countries, especially those with the highest pneumonia burden. Despite such hopes, the peer-reviewed literature on PCV-related mortality reduction is scarce. We searched PubMed, Google Scholar, and Scielo for peer-reviewed post-marketing studies on the effect of PCVs on pneumonia mortality using the terms “pneumonia”, “pneumococcal vaccine/s”, “conjugate vaccine/s”, “PCV”, “pneumococcal”, “impact”, and “effectiveness”. We included studies analysing changes in pneumonia mortality data before and after PCV introduction for any paediatric age group up to Dec 31, 2017, published in English, Spanish, and Portuguese, and excluded studies limited to in-hospital mortality. We identified only five post-marketing effectiveness studies; of these, three were set in a subnational population. Results were inconsistent, with benefits ranging from negligible to very large. Therefore, we set out to assess the effect of ten-valent pneumococcal conjugate vaccine (PCV10) on childhood pneumonia mortality in Brazil, a large country with high national vaccine coverage and substantial disparity in wealth, allowing comparisons of effectiveness among children from different socioeconomic groups.

**Added value of this study**

Brazil added PCV10 to its national vaccine schedule in 2010. We found that between 1980 and 2010, childhood pneumonia mortality decreased by 10 times in Brazil, concomitant with socioeconomic developments that led to improved education, sanitation, and public health. Regarding the effect of PCV10, we found weak evidence that introducing the vaccine produced a further reduction in childhood pneumonia deaths of about 10% at the national level. After stratifying by socioeconomic status, we found larger reductions in the subpopulation living in municipalities characterised by poverty or low maternal education, but no decline in the more affluent municipalities.

**Implications of all the available evidence**

Although a vaccine-related decrease in childhood pneumonia mortality of 10% is less than what many expected, our estimates indicate that the vaccine had a substantially greater benefit in poor municipalities in Brazil. These results imply that although the effect of PCV on paediatric pneumonia mortality might be reduced in countries where mortality rates have already fallen because of economic and social improvements, substantial vaccine-related gains are more likely in the world's low-income regions. Because reliable vital statistics data are largely unavailable in these countries, where pneumonia mortality is still high, assessing these gains will be difficult.

However, preventing pneumonia deaths among children is the fundamental goal of the global effort to introduce PCVs. For example, WHO estimates that PCV use could reduce global childhood pneumonia mortality by up to 30%^8^ and urges PCVs to be a priority in all countries, especially in those with the highest mortality in children younger than 5 years.^9^ Although it stands to reason that fewer children will die from pneumonia if fewer children fall ill with pneumonia, surprisingly few published studies have estimated the benefit of PCVs for childhood pneumonia mortality after PCVs have been introduced.

Therefore, we set out to assess the effect of PCV10 introduction on pneumonia mortality in children in Brazil. Brazil is a promising setting for such an assessment because PCV10 coverage rose rapidly after its 2010 introduction, meaning benefits should become apparent quickly; long time series of detailed record-level vital statistics data are available; and Brazil has substantial geographical disparity in wealth, allowing the comparison of effectiveness among children from different socioeconomic groups. Our analysis should shed light on PCV benefits in poor countries where the pneumonia burden is large, but vital statistics data are largely unavailable.

## Methods

### Study design and data sources

We accessed publicly available mortality data of children aged 59 months or younger between 1980 and 2014, coded according to the International Classification of Diseases tenth edition (ICD-10), from the Mortality Information System of the Brazilian Ministry of Health.^10^ We separated data into three age groups for analysis: children aged 3–11 months, 3–23 months, and 3–59 months. Infants aged 0–2 months were excluded to avoid perinatal causes of mortality—a major source of childhood mortality in Brazil^11^ not preventable by PCV. Data on births and deaths are systematically collected throughout the year in Brazil, and covered approximately 95% of the population in 2010.^12^ We assessed all-cause pneumonia for all analyses, defined as ICD-10 codes J12–18, as the primary cause of death; these represented 65% of all respiratory deaths (ICD10 J00–99) among children in 2010.^10^

**Figure 1:**
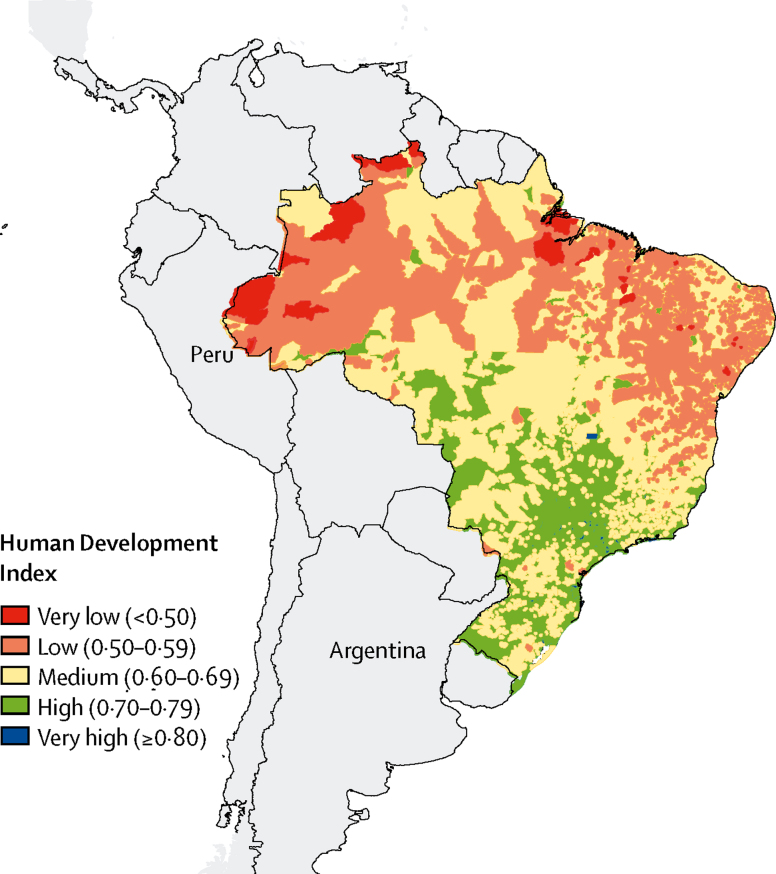
Geographic distribution of 5770 Brazilian municipalities in 2010, on the basis of their Human Development Index

We stratified Brazil's 5570 municipalities into low, medium, and high socioeconomic status (SES) categories in three distinct ways, on the basis of data from 2010. We first stratified municipalities by their Human Development Index (HDI)^13^—a composite measure of income, education, and longevity—into the categories very low or low (<0·60), medium (0·60 to <0·70), and high or very high development (≥0·70). The geographical distribution is shown in figure 1. Because vulnerability to pneumonia and access to treatment can vary with other SES factors not easily captured by a general index such as HDI, we also stratified municipalities by the proportion of children living in extreme poverty (monthly per capita income <US$40)^14^ and the proportion of mothers with no primary education;^15^ both of which have been recognised as important risk factors for severe pneumonia in children.^16^ For percentage of children in poverty, we defined low SES as more than 27% of children from a municipality living in extreme poverty, and high SES as fewer than 3% of children living in extreme poverty (upper and lower quartiles of extreme poverty distribution). For maternal education, we defined low SES as more than 30% of mothers with no primary education in a municipality, and high SES as fewer than 10% of mothers without primary education (upper and lower quartiles of the distribution).

Population estimates for each municipality and age group were obtained from the Brazilian Institute of Geography and Statistics (censuses 1991, 2000, and 2010). Monthly population data for SES strata were calculated by cubic polynomial interpolation of the age-stratified and municipality-stratified census data with Popweaver,^17^ a freely available interpolation software package.

We obtained PCV10 coverage data from the National Immunisation Programme.^18^ The denominator for vaccine uptake was the number of children in each respective age band, estimated from monthly municipality-specific livebirth data.^19^ We used a cohort model that computes coverage in ageing cohorts of vaccinated infants from the numbers of doses administered in the post-vaccination period^20^ to estimate the proportion of children aged 6–23 months who received 3 or more doses of PCV10 or received the age-appropriate vaccine over time (eg, one catch-up dose after age 1 year if not eligible for full series).

### Procedures

The pre-vaccine period was from April 1, 2004, to March 31, 2009 (5 full years, ensuring that all seasons were equally represented), and the post-vaccine period was from April 1, 2010, to March 31, 2014 (4 full years). The 2009 influenza virus H1N1 pandemic affected Brazil from April, 2009, to March, 2010; therefore, this period was excluded from all analyses. Because it took approximately 2 years for PCV10 to reach full coverage, rate ratios were calculated for the period April 1, 2012, to March 31, 2014. Additionally, because sparse data tend to be noisier, mortality analyses were based on quarterly instead of monthly time series (Q1: January–March, Q2: April–June, Q3: July–September, Q4: October–December).

For each age group and demographic stratum, we compiled time series of pneumonia deaths per quarter. We used two complementary regression methods (synthetic control analysis and time-trend adjustment) to assess changes in pneumonia deaths after the introduction of PCV10. In both methods, the models were fit to data from the pre-vaccine period alone and controlled for seasonal variations with quarterly dummy variables. The regression models were then used to project how many cases of pneumonia would have occurred in the absence of PCV10 during the post-vaccine period. Rate ratios were calculated by comparing observed and counterfactual rates in the post-PCV10 period. In both methods, the data were fit in a Bayesian setting that allowed us to quantify uncertainty associated with variable selection and variation in the data. The approaches differed in how they adjust for changes unrelated to vaccine introduction.

We assessed the effect of PCV10 introduction on childhood pneumonia mortality, both at the national level and in high, middle, and low SES groups of municipalities.

### Statistical analysis

We have previously described the synthetic control analysis method in the context of assessing the effect of PCV10 on pneumonia hospitalisations.^21^ With this method, categories of mortality that were not expected to be influenced by the introduction of PCV10 were used as control variables in a regression. A time series for each control variable was constructed, and the scaled, log-transformed version of the time series was used as a covariate. Rather than using forwards or backwards variable selection, synthetic control analysis used a Bayesian variable selection method to determine the contribution of each possible control. Control time series that resembled the pneumonia time series more closely had higher weights, and irrelevant time series received weights closer to zero. These weights and regression coefficients were then combined with the observed values for the control time series in the post-vaccine period to generate a counterfactual estimate.

In this study, we applied the synthetic control method^21^ with a few adaptations. To construct the synthetic control, we used time series defined by ICD-10 subchapters, in which disease codes are grouped in epidemiologically meaningful categories. We excluded subchapters containing codes that might be affected by PCVs from the synthetic control: all respiratory (J) subchapters; other bacterial diseases (A30–49); diseases of the middle ear and mastoid process (H65–75); bacterial, viral, and other infectious agents (B95–98); and inflammatory diseases of the central nervous system (G00–09).

In addition to the synthetic control analysis, we did a time-trend adjustment. We fit a simpler regression model in which the only control variables were a linear trend for time and quarterly dummy variables to adjust for seasonality; the offset term was all deaths that PCV10 could not be expected to prevent (non-PCV10-preventable deaths). Because we included an offset for all non-pneumococcal deaths, the interpretation of this model is effectively the extent to which pneumonia deaths changed relative to all other non-pneumococcal-related causes. With this commonly used time-trend model, the counterfactual in the post-vaccine period was generated by assuming that the pre-vaccine trends and seasonality continued into the post-vaccine period. Non-PCV-preventable deaths were defined as all deaths except those coded as respiratory (any ICD-10 J code), otitis media (H65–66), sepsis (A40–41), meningitis (G00–03 and A39), unspecified bacterial infection (A49), and encephalitis (G04).

We did two validation analyses. In the first analysis, because completeness of vital registration data increased over time, we investigated whether adjusting for underreporting^22^ would change our estimates. On the basis of data from the Brazilian Ministry of Health (2015), in which ill defined causes of death were proportionally redistributed on the basis of age, sex, Brazilian state of residence, and the relative proportion of deaths in each ICD-10 chapter, we estimated correction factors for each age group, ICD-10 chapter, year, and state as the ratio of adjusted to unadjusted mortality. We then used this ratio to adjust the time series of the outcome of interest (J12–18) and the ICD-10 subchapters used to create the synthetic control. For the second validation analysis, we applied the synthetic control method to data from the period before PCV-10 was introduced, defining April, 2004, to March, 2007, as the training period and April, 2007, to March, 2009, as the evaluation period.

We used the R statistical analysis software package for all analyses. The R code and mortality time series data can be downloaded from GitHub.

### Role of the funding source

The funder of the study had no role in study design, data collection, data analysis, data interpretation, or writing of the report. All authors had full access to all the data and fully agreed with the decision to submit the paper for publication. The corresponding author had final responsibility for the decision to submit for publication.

## Results

**Figure 2:**
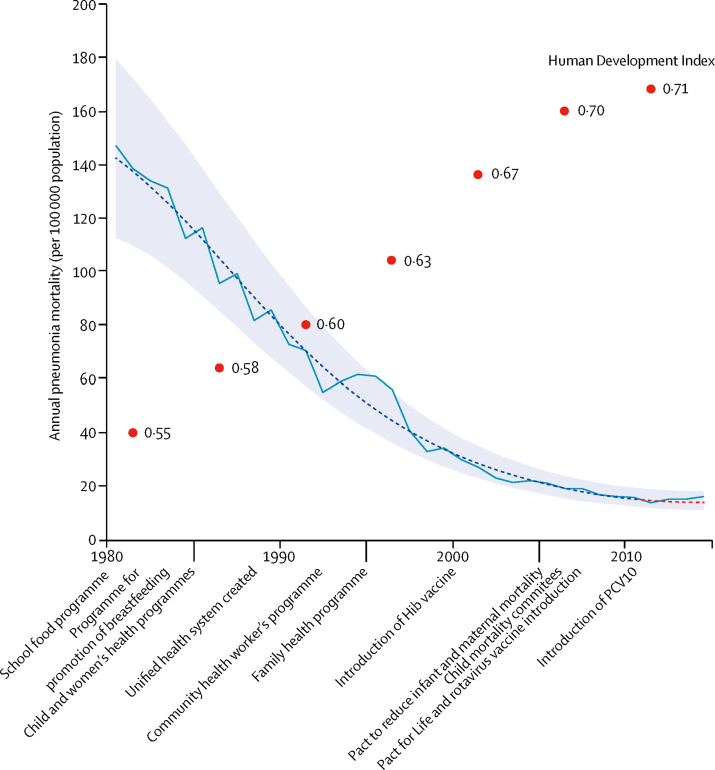
National annual all-cause pneumonia mortality rate from 1980 to 2014 in children younger than 5 years and Human Development Index evolution over time in Brazil National annual all-cause pneumonia mortality rate from 1980 to 2014 is represented by a solid line, and the Human Development Index evolution over time is represented by red dots. An exponential fit to the data is shown as a dashed line, with associated 95% CIs shown as shaded area. Selected national programmes for improving childhood health and reducing mortality are indicated: National School Food Programme in 1979, National Programme for the Promotion of Breastfeeding in 1981, National Programmes for Child Health and for Women's Health in 1984, creation of the Unified Health System in 1988, Community Health Workers Programme in 1991, Family Health Programme to increase access to health care in Brazil's poorest areas in 1994, introduction of *Haemophilus influenzae* (Hib) vaccine in 2000; Pact for Reduction of Maternal and Newborn Mortality in 2004, creation of local committees for prevention of infant mortality in 2005, Pact for Life (goals included reduction of infant deaths by diarrhoea by 50% and pneumonia by 20%) in 2006, introduction of rotavirus vaccine in 2006, and introduction of ten-valent pneumococcal conjugate vaccine (PCV10) in 2010.

Pneumonia mortality in Brazilian children decreased by about 90% during 1980–2010, dropping from 147·0 to 16·1 annual deaths per 100 000 children younger than 5 years (figure 2). During these pre-PCV decades, Brazil transitioned from a lower-middle-income to a high-middle-income economy, and its HDI increased from 0·55 to 0·71. Reductions in annual rates of pneumonia mortality were steepest in the 1980s and 1990s. Therefore, most of the decline since 1980 was achieved before the millennium and preceded the introductions of the *Haemophilus influenzae* type B vaccine in 2000, and PCV10 in 2010.

**Figure 3:**
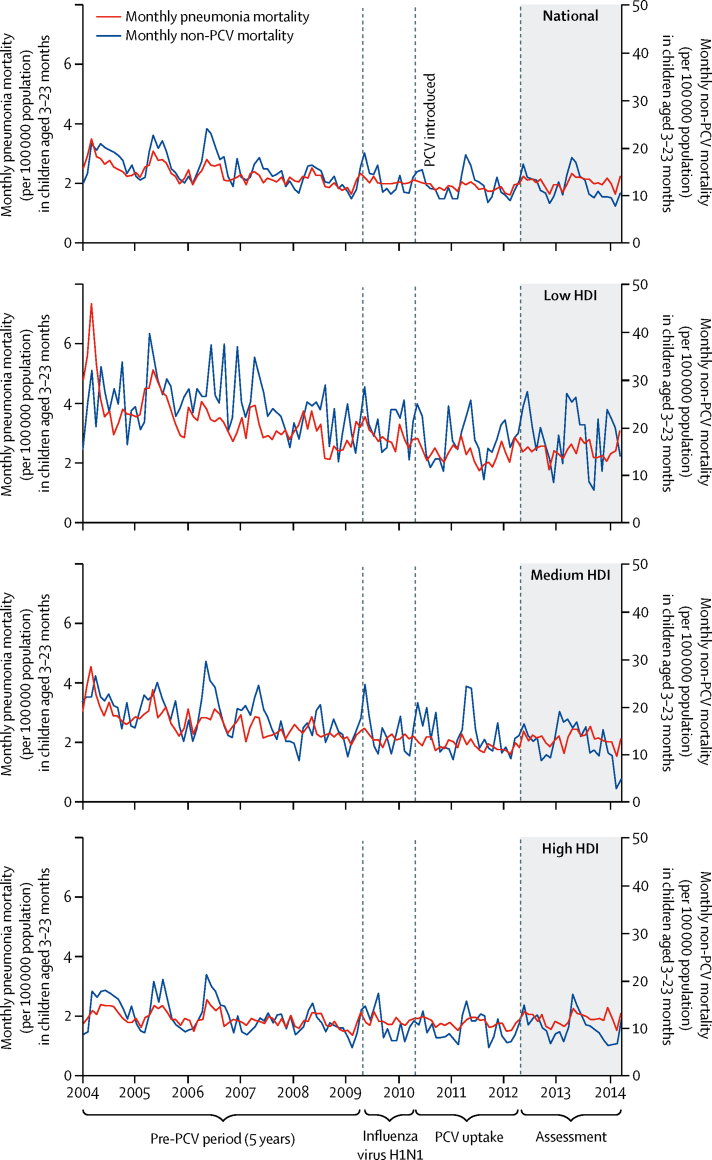
Time series of pneumonia monthly mortality (per 100 000 population) among children aged 3–23 months, nationally and stratified by Human Development Index (HDI) Non-PCV mortality=mortality not preventable by PCV use. PCV=pneumococcal conjugate vaccine.

We assessed raw trends in the years immediately before (2004–09) and after the 2010 introduction of PCV10 using both national data and data from Brazil's 5750 municipalities stratified by SES. During this period, national monthly pneumonia mortality rates declined modestly, but were essentially flat and continued to be flat thereafter (figure 2). A similar pattern was observed for non-PCV-preventable deaths (all deaths except those potentially affected by PCV10 introduction; appendix). We found that the low SES strata—on the basis of HDI, proportion of children living under extreme poverty, and proportion of mothers without primary school education—had higher rates of pneumonia mortality at the time of PCV10 introduction in 2010 than those of the high SES strata (table 1). Time series of pneumonia mortality rates in municipalities stratified by any of the three levels of HDI did not visibly change after 2010 (figure 3; appendix). PCV10 uptake was lower in the lower SES groups than in the higher SES groups during the first 2 post-PCV years, but these differences faded by mid-2012, as PCV10 coverage reached high levels (80–85% of the target population) in all strata (appendix).

**Table 1:** Pneumonia mortality per 100 000 population in Brazilian children in 2010 by age group, nationally and by socioeconomic indicators

		**3–11 months**			**3–23 months**			**3–59 months**		
		Mortality (per 100 000)	Number of deaths	Population (in thousands)	Mortality (per 100 000)	Number of deaths	Population (in thousands)	Mortality (per 100 000)	Number of deaths	Population (in thousands)
National		37	746	2035	24	1126	4730	11	1459	13 118
Human Development Index										
	Low	58	167	288	36	244	683	15	295	1944
	Medium	42	197	473	29	321	1106	13	413	3097
	High	30	382	1274	19	561	2941	9	751	8077
Poverty*										
	Low	55	168	305	35	255	720	15	313	2052
	Medium	41	392	961	26	589	2241	12	752	6217
	High	24	186	768	16	282	1766	8	394	4843
Maternal education†										
	Low	53	113	213	36	180	504	16	223	1430
	Medium	36	591	1637	23	886	3801	11	1152	10 521
	High	23	42	184	14	60	425	7	84	1167

* On the basis of percentage of children in extreme poverty.

† On the basis of percentage of mothers without education.

**Figure 4:**
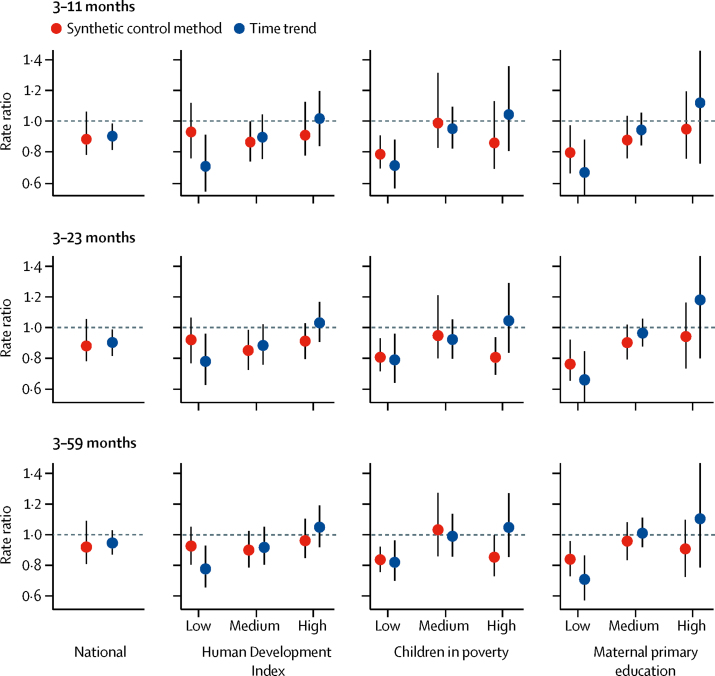
Changes in pneumonia mortality in three paediatric age groups associated with pneumococcal conjugate vaccine introduction in Brazil Analyses are presented nationally and stratified by Human Development Index, percentage of children living in extreme poverty, and percentage of mothers without primary school education.

We assessed declines in childhood pneumonia mortality associated with PCV10 use, adjusting for non-vaccine-related trends with the synthetic control method. At the national level, the point estimates of the rate ratios were about 0·9 in the three age groups, corresponding to a reduction of about 10% (figure 4, appendix). These estimates had substantial uncertainty, and all credible intervals (CrIs) included 1 (signifying no effect): the estimated reduction was 12% (95% CrI −6 to 12) for children aged 3–11 months, 12% (–6 to 13) for children aged 3–23 months, and 8% (–9 to 19) for children aged 3–59 months. However, we detected a pattern of larger decreases in post-PCV10 pneumonia mortality—decreases with 1 outside the CrI—in all three paediatric age groups in municipalities with a high percentage of extreme childhood poverty and mothers with no primary education (figure 4). For example, in the 3–23 months group, the mortality reduction was 19% (95% CrI 7–28) in high-poverty municipalities and 24% (7–35) in municipalities with low maternal education (figure 4; appendix). This pattern of decreases was not present when we stratified by HDI, where the middle stratum had the largest estimated decrease. A consistent trend towards larger mortality decreases among successively less privileged populations in all three age groups was clear when SES was stratified by maternal primary education.

In addition to the primary synthetic control analysis, we did a simpler time-trends adjustment. We observed a pattern of results similar to the primary analysis, but with narrower CrIs. As a result, the estimated declines in pneumonia mortality in all three low-SES status strata and in all age groups had the upper bound of the CrIs below 1 (figure 4; appendix).

As a validation of the primary analysis, we applied the synthetic control model to a time period when no vaccine effect would be expected; we used April, 2004–March, 2007, as the training period and April, 2007–March, 2009, as the evaluation period. Most point estimates were lower than 1, and the three low-SES strata had CrIs that were below 1. This potentially indicates uncontrolled confounding in these strata. However, CrIs for the rate ratios of most strata included 1 (appendix).

We investigated the effect of adjusting the data for under-reporting of deaths and found that doing so made no difference in the point estimates or CrIs produced in either synthetic control or time-trend analyses and did not affect any of the observed trends or conclusions.

## Discussion

In this study, we observed a large decrease in childhood pneumonia mortality in Brazil between 1980 and 2010, concomitant with the rapid socioeconomic development of the country and probably driven by many factors, including improved nutrition, housing, education, and access to health care, especially antibiotic drugs. We assessed the effect of the introduction of PCV10 in 2010, at national and municipality levels and controlling for continuing trends associated with development. With two different statistical models, we found modest evidence for a reduction in national pneumonia mortality in the two age groups with children younger than 24 months. Because this was a population-level estimate, it captured the effects of both serotype replacement, which would tend to reduce the benefit observed, and herd immunity, which would tend to increase it.

How do these national estimates compare with expectations? WHO has stated that childhood pneumonia mortality could be reduced by 30% if PCVs were used everywhere.^8^ A frequently cited randomised controlled trial^23^ of nine-valent PCV done in The Gambia in the early 2000s found, in a post-hoc analysis, that the vaccine reduced all-cause childhood mortality by 16%—a far broader endpoint and, thus, a much larger benefit than the reduction that WHO states could be achieved in pneumonia mortality alone. Estimates from the handful of published post-introduction studies of the effect of PCV use on childhood pneumonia mortality are variable, although the benefits are generally larger than those we found in Brazil; one study from Brazil^24^ reported no benefit, whereas another from Chile^25^ estimated an extraordinarily large benefit (table 2).

**Table 2:** Published post-marketing studies on the effect of pneumococcal conjugate vaccines (PCVs) on pneumonia and randomised controlled trials assessing the effect of PCVs on mortality in children

	**Vaccine**	**Age (months)**	**Period**	**Method**	**Adjusted for pre-vaccine trends?**	**Control outcome**	Mortality outcome*	**% Reduction**†**(95% CI)**	**Mortality outcome**	**Events (controls)**	**Events (vaccine)**	**Relative risk (95% CI)**
**Observational post-licensure studies**												
Brazil, Santa Catarina^24^	PCV10	<12	2006–13	Pre-post	Yes	No	J13, J15, J18	11% (−20 to 34)	NA	NA	NA	NA
Chile^25^	PCV10	2–23	2010–11	Case-control	NA	No	J13–J18	72% (9 to 92)	NA	NA	NA	NA
Nicaragua, León^26^	PCV13	<12	2008–12	Pre-post	No	Diarrhoea	Clinical pneumonia	33% (20 to 43)	NA	NA	NA	NA
Nicaragua, León^27^	PCV13	<12	2008–15	Pre-post	No	Diarrhoea	Clinical pneumonia	44% (23 to 59)	NA	NA	NA	NA
Peru^28^	PCV10	<12	2006–12	Pre-post	Yes	No	J12–J18	35% (9 to 54)	NA	NA	NA	NA
**Randomised controlled trials**												
South Africa^29^	PCV9	<24	NA	NA	NA	NA		NA	LRI; all cause	LRI 160 (19 914); all cause 242 (19 914)	LRI 161 (19 992); all cause 229 (19 992)	LRI 1·01 (0·76–1·2); all cause 0·95 (0·79–1·13)
The Gambia^23^	PCV9	3–29	NA	NA	NA	NA		NA	All cause	389 (8151)	330 (8189)	0·84 (0·72–0·97)
Latin America^30^	PCV10	<24	NA	NA	NA	NA		NA	All cause	26 (11 799)	19 (11 798)	0·73 (0·41–1·32)

The number following PCV indicates the number of serotypes covered by the vaccine. Pre-post=a study that compared rates in the pre-vaccine period to rates in the post-vaccine period. NA=not applicable. LRI=lower respiratory tract infection.

* Codes of the International Classification of Diseases tenth edition.

† Some authors based the estimate on last year in study, others on more than 1 post-PCV years.

Although we only detected a modest reduction in childhood pneumonia mortality at the national level after PCV10 introduction, we reported more substantial declines in less privileged populations, with point estimates of the vaccine-associated decline of 16–24% among the smaller subset of children from areas with a high proportion of mothers with low education. Several factors could explain the discrepant patterns between children from higher SES and lower SES. For example, higher SES might be associated with changes in the proportion of pneumonia caused by pneumococcus or with better diagnosis and access to treatment. Our results suggest that in low-income countries, where the paediatric pneumonia mortality burden is larger and timely access to care and maternal knowledge about disease are scarcer than in developed countries, the mortality reduction after introduction of PCVs might be larger than in higher-income nations.

Our study has limitations. Although the synthetic control method we used to detect the benefits of PCV introduction was explicitly designed to minimise confounding, the method is, at heart, an ecological study design and other uncontrolled factors might have affected the estimates. For example, it is possible that our estimates were confounded by antibiotics becoming prescription-only drugs in Brazil in the same year that PCV10 was introduced.^31^ Moreover, we relied on ICD-10 codes being mostly accurately assigned, and we assumed that no substantial changes in the probability of miscoding happened over time. However, our study assessed all paediatric deaths in the entire country over multiple years; to the extent that PCV10 indeed had a substantial effect on childhood pneumonia mortality, we were probably able to estimate it accurately.

The massive reduction in childhood pneumonia mortality observed in Brazil after 1980, and before the introduction of PCV10, underscores the often underappreciated power of improved nutrition, health care, education, and living conditions to save lives. Although we cannot know what the effect of PCV10 might have been in Brazil had it been introduced in 1980, our SES-stratified results suggest that the effect would have been considerably greater than it was in 2010. To the extent that Brazil's poorer regions can act as a proxy for low-income countries elsewhere, our study suggests that the power of PCVs to save children's lives is likely to be greatest in countries that are less economically developed, and where the burden of pneumonia mortality is still high.
