# Supplementary material for: Effect of pneumococcal conjugate vaccine introduction on childhood pneumonia mortality in Brazil: a retrospective observational study
Source: Lancet Glob Health. 2019 Jan 22;7(2):e249–56. doi: 10.1016/S2214-109X(18)30455-8 (PMC6344339; doi:10.1016/S2214-109X(18)30455-8)
Supplement: Supplementary appendix [file mmc1.pdf]

THE LANCET
Global Health

Supplementary appendix

This appendix formed part of the original submission and has been peer reviewed. We post it as supplied by the authors.

Supplement to: Schuck-Paim C, Taylor RJ, Alonso WJ, Weinberger DM, Simonsen L. Effect of pneumococcal conjugate vaccine introduction on childhood pneumonia mortality in Brazil: a retrospective observational study. *Lancet Glob Health* 2019; **7:** e249–56.

Supplement to Schuck-Paim C, Taylor RJ, Alonso WJ, Weinberger DM, Simonsen L. Effect of pneumococcal conjugate vaccine introduction on childhood pneumonia mortality in Brazil: a retrospective observational study.

SUPPLEMENTARY TABLES

Table S1. Synthetic Control method, estimate rate ratios with and without PCV use.

	SES	3-11m	3-23m	3-59m
**National**		.88(.78, 1.06)	0.88(.77, 1.06)	0.92(.81, 1.09)
Human Development Index	Low	.93(.76, 1.13)	.92(.76, 1.06)	.94(.81, 1.06)
	Medium	.87(.74, 1.00)	.85(.72, .98)	.91(.79, 1.03)
	High	.92(.78, 1.14)	.90(.79, 1.03)	.97(.86, 1.11)
Poverty (% children in extreme poverty)	Low	.77(.68, .90)	.81(.72, .93)	.84(.76, .93)
	Medium	.98(.81, 1.32)	.95(.80, 1.21)	1.03(.86, 1.27)
	High	.85(.68, 1.13)	.81(.69, .94)	.86(.74, 1.00)
Maternal Education (% mothers with limited education)	Low	.79(.67, .98)	.76(.65, .93)	.84(.73, .96)
	Medium	.88(.76, 1.04)	.90(.79, 1.03)	.95(.83, 1.07)
	High	.95(.76, 1.20)	.94(.74, 1.17)	.90(.72, 1.09)

Table S2. Time Trends method estimated rate ratios with and without PCV use.

**Strata**	**SES**	**3-11mo**	**3-23mo**	**3-59mo**
**National**		.90(.82, .99)	.90(.82, .98)	.95(.87, 1.03)
Human Development Index	Low	.72(.56, .93)	.77(.62, .95)	.78 (.66-.93)
	Medium	.91(.77, 1.06)	.87(.75, 1.00)	.93 (.81-1.06)
	High	1.02(.86, 1.21)	1.01(.89, 1.15)	1.05 (.92-1.2)
Poverty (% children in extreme poverty)	Low	.71(.57, .89)	.79(.64, .96)	.82(.70, .96)
	Medium	.96(.83, 1.11)	.92(.80, 1.06)	.99(.86, 1.14)
	High	1.06(.81, 1.38)	1.04(.84, 1.29)	1.05(.86, 1.27)
Maternal Education (% mothers with limited education)	Low	.67(.51, .89)	.65(.51, .83)	.70(.57, .86)
	Medium	.95(.85, 1.06)	.95(.86, 1.05)	1.00(.91, 1.10)
	High	1.12(.73, 1.70)	1.17(.79, 1.72)	1.10(.78, 1.55)

Table S3. Primary contributors to the synthetic control by age group and stratum.

Strata	Age	Level	Control 1	Control 2	Control 3
National	3-<12m		NoPCV	A00_A09	R95_R99
	3-<24m		NoPCV	A00_A09	R95_R99
	3-<60m		NoPCV	R95_R99	A00_A09
HDI	3-<12m	Low	A00_A09	E40_E46	NoPCV
		Medium	NoPCV	R95_R99	E40_E46
		High	NoPCV	A00_A09	E40_E46
	3-<24m	Low	A00_A09	E40_E46	NoPCV
		Medium	NoPCV	R95_R99	E40_E46
		High	NoPCV	R95_R99	A00_A09
	3-<60m	Low	A00_A09	E40_E46	NoPCV
		Medium	NoPCV	E40_E46	R95_R99
		High	NoPCV	R95_R99	A00_A09
Child Poverty	3-<12m	Low	E40_E46	NoPCV	A00_A09
		Medium	NoPCV	A00_A09	R95_R99
		High	NoPCV	E40_E46	R95_R99
	3-<24m	Low	E40_E46	NoPCV	A00_A09
		Medium	NoPCV	A00_A09	R95_R99
		High	NoPCV	R95_R99	V01_X59
	3-<60m	Low	E40_E46	NoPCV	A00_A09
		Medium	NoPCV	A00_A09	R95_R99
		High	R95_R99	NoPCV	V01_X59
Maternal education	3-<12m	Low	E40_E46	A00_A09	NoPCV
		Medium	NoPCV	R95_R99	A00_A09
		High	A00_A09	V01_X59	R95_R99
	3 -<24m	Low	E40_E46	A00_A09	NoPCV
		Medium	NoPCV	R95_R99	A00_A09
		High	A00_A09	NoPCV	Q20_Q28
	3-<60m	Low	A00_A09	E40_E46	NoPCV
		Medium	NoPCV	R95_R99	V01_X59
		High	A00_A09	R95_R99	NoPCV

SUPPLEMENTARY FIGURES

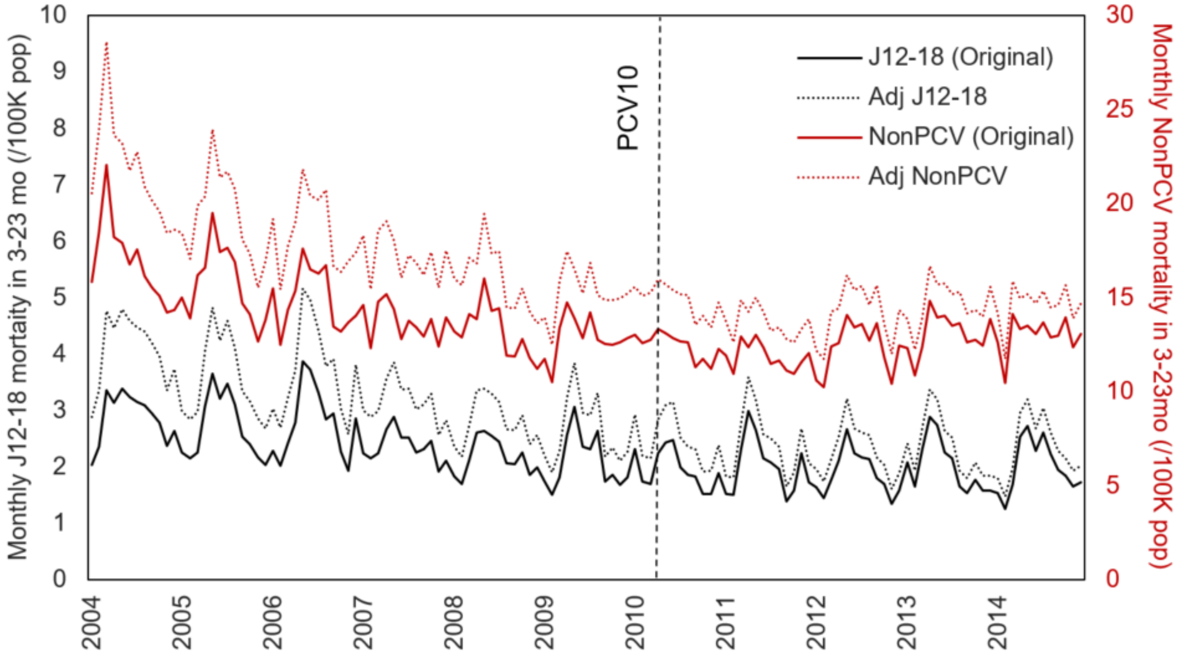

Figure S1. Time series of monthly mortality rates (deaths/100,000 population) from pneumonia (lower pair of lines) and all deaths except respiratory, sepsis and meningitis (Non-PCV deaths, upper pair) among children 3-23 months. The dotted line is adjusted for underreporting of deaths, the solid line is not. The vertical dashed line indicates the time of PCV introduction.

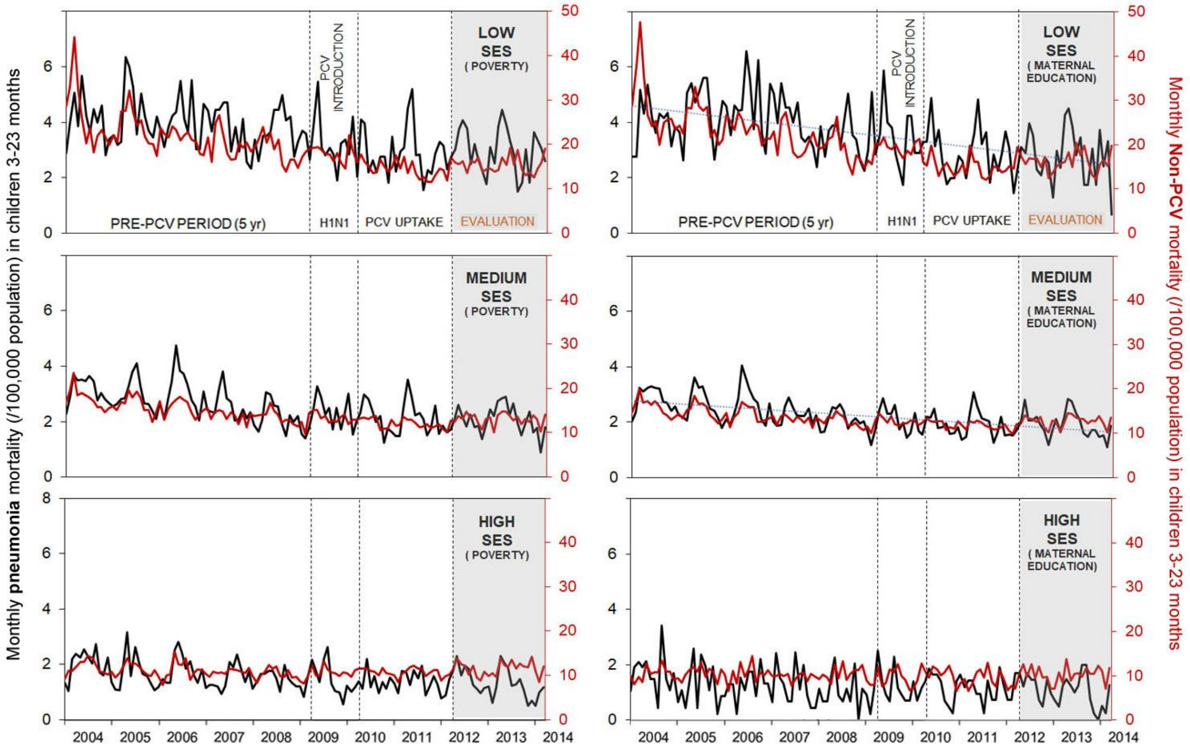

Figure S2. Time series of pneumonia deaths/100,000 population among children 3-29 months of age, stratified by percent of children living in extreme poverty (left) and percent of mothers without primary school education (right). The vertical dashed line indicates the time of PCV introduction.

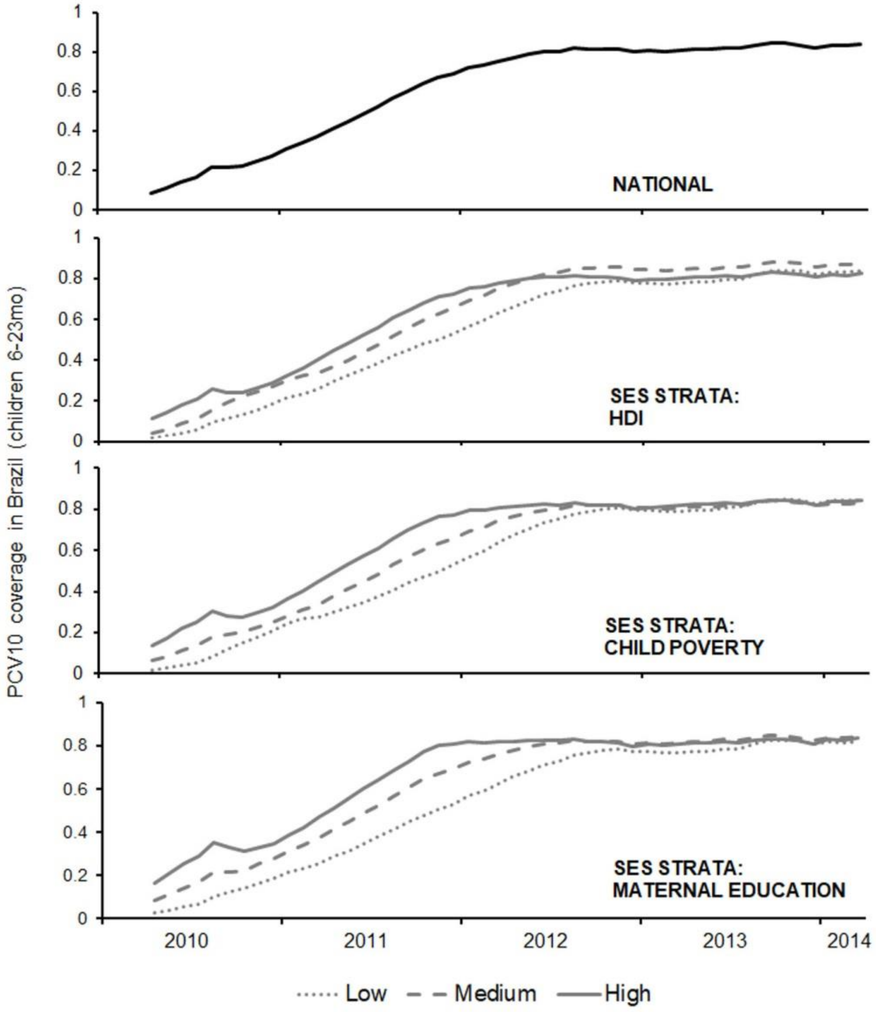

Figure S3. Estimated percentage of Brazilian children fully immunized with PCV10, or who received a catch-up dose of PCV10 for each of the socioeconomic stratifications studied (HDI: human development index; Child Poverty: municipalities with low, medium or high percentages of children living under extreme poverty; Maternal education: municipalities SES scores based on the proportion of mothers without primary school education).

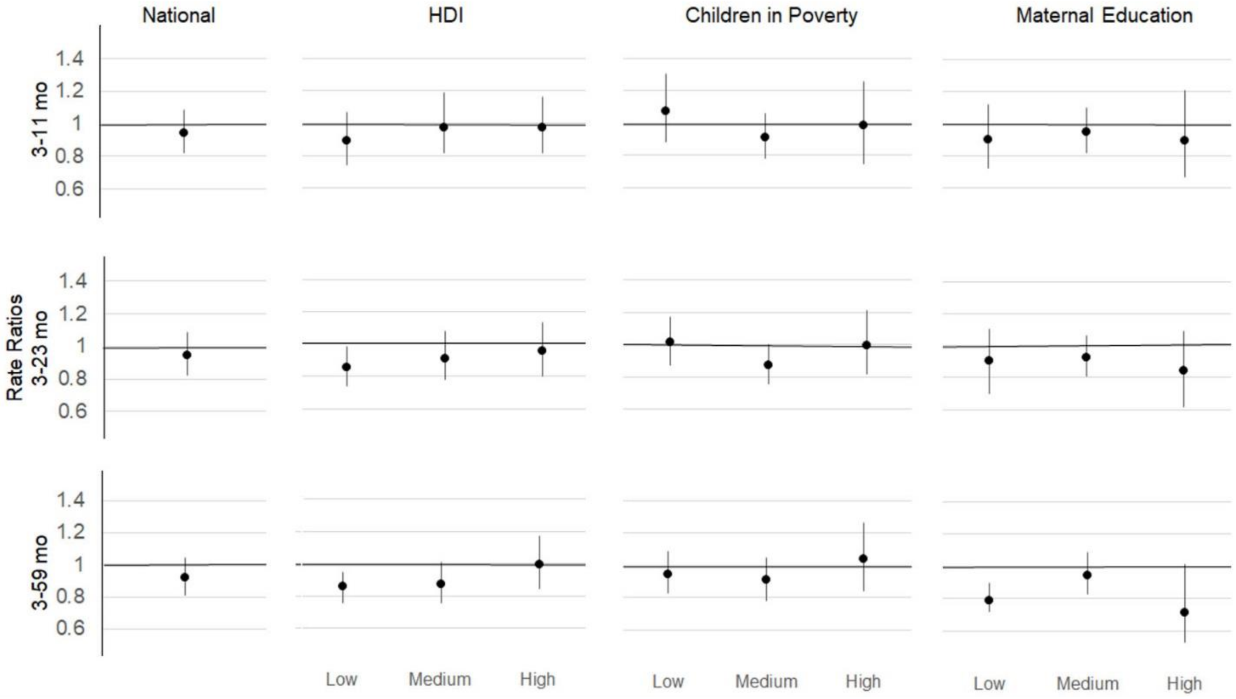

Figure S4. Validation analysis in which both the training (April 2004 to March 2007) and evaluation period (April 2007 to March 2009) were set before PCV10 introduction in Brazil; rate ratios given for three pediatric age groups nationally and stratified by three indicators of socioeconomic status (Human Development Index, percent of children living in extreme poverty, and percent of mothers without primary school education).
